# Summary of available surveillance data on hepatitis C virus infection from eight Arctic countries, 2012 to 2014

**DOI:** 10.2807/1560-7917.ES.2018.23.40.1700408

**Published:** 2018-10-04

**Authors:** Prabhu P Gounder, Anders Koch, Ginger Provo, Astrid Lovlie, Josefine Lundberg Ederth, Maria Axelsson, Chris P Archibald, Brendan Hanley, Angie Mullen, Myrna Matheson, David Allison, Henrik Trykker, Thomas W Hennessy, Markku Kuusi, Vladimir Chulanov, Brian J McMahon

**Affiliations:** 1 Arctic Investigations Program, Division of Preparedness and Emerging Infections, National Center for Emerging and Zoonotic Infectious Diseases, U.S. Centers for Disease Control and Prevention (CDC), Anchorage, Alaska, USA; 2 Statens Serum Institut, Copenhagen, Denmark; 3 Lisimatusarfik, University of Greenland, Nuuk, Greenland; 4 Division of Public Health, State of Alaska, Anchorage, Alaska, USA; 5 Department for Infectious Disease Registries, Norwegian Institute of Public Health, Oslo, Norway; 6 Department of Public Health Analysis and Data Management, Public Health Agency of Sweden, Stockholm, Sweden; 7 Unit for Epidemiology & Health Economic, the Public Health Agency of Sweden, Stockholm, Sweden; 8 Surveillance and Epidemiology Division, Centre for Communicable Diseases and Infection Control, Public Health Agency of Canada, Ottawa, Canada; 9 Chief Medical Officer of Health, Whitehorse, Yukon, Canada; 10 Department of Health, Government of Nunavut, Iqaluit, Nunavut, Canada; 11 Communicable Disease Control Unit, Department of Health and Social Services, Government of the Northwest Territories, Yellowknife, Northwest Territories, Canada; 12 Department of Health and Community Services, St. John’s, Newfoundland and Labrador, Canada; 13 National Board of Health, Nuuk, Greenland; 14 National Institute for Health and Welfare (THL), Helsinki, Finland; 15 Central Research Institute of Epidemiology, Reference Center for Viral Hepatitis, Moscow, Russia; 16 Liver Diseases and Hepatitis Program, Alaska Native Tribal Health Consortium, Anchorage, Alaska, USA

**Keywords:** viral hepatitis, surveillance, prevention and control, indigenous populations, North America, northern Europe

## Abstract

We summarised available hepatitis C virus (HCV) surveillance data for 2012–14 from Arctic/sub-Arctic countries/regions. We sent a HCV data collection template by email to public health authorities in all jurisdictions. Population statistics obtained from census sources for each country were used to estimate rates of reported acute and chronic/undifferentiated HCV cases. Seven countries with Arctic regions (Canada, Denmark, Finland, Greenland, Norway, Sweden and the United States, represented by the state of Alaska), including three Canadian territories and one province, as well as 11 Russian subnational Arctic regions, completed the data collection template. Data on acute HCV infection during 2014 was available from three Arctic countries and all Russian Arctic regions (rate range 0/100,000 population in Greenland, as well as Nenets and Chukotka Automous Okrugs (Russian subnational Arctic regions) to 3.7/100,000 in the Russian Republic of Komi). The rate of people with chronic/undifferentiated HCV infection in 2014 ranged from 0/100,000 in Greenland to 171.2/100,000 in Alaska. In most countries/regions, the majority of HCV-infected people were male and aged 19–64 years. Differences in surveillance methods preclude direct comparisons of HCV surveillance data between Arctic countries/regions. Our data can inform future efforts to develop standardised approaches to HCV surveillance in the Arctic countries/regions by identifying similarities/differences between the surveillance data collected.

Although hepatitis C virus (HCV) is a major public health concern in many countries, the precise incidence and prevalence of HCV infections are unknown in large parts of the world [1]. The burden of HCV infection is difficult to ascertain because many HCV-infected people are asymptomatic or those at risk might not be tested for HCV infection. Furthermore, many widely used screening tests do not distinguish current from resolved infection or people identified with HCV infection might not be reported to public health authorities. To overcome these limitations, statistical models have been developed to provide more comprehensive estimates of the prevalence of HCV infection [2,3]. According to one recent model, ca 115 million people (prevalence: 1.6%) worldwide had serologic evidence of HCV infection (anti-HCV antibody-positive) in 2013, including 80 million people (prevalence: 1.1%) with ongoing HCV viraemia [3].

Over four million people live in the Arctic region [4,5], although the precise boundary of the Arctic is debatable. For the purposes of this report, we used the Arctic Human Development Report’s definition of the Arctic boundary [5]. Countries and regions with populations in the Arctic include the United States (US), represented by Alaska (the only Arctic state); Canadian territories north of 60°N (Yukon, Nunavut and Northwest Territories), plus the northern province of Newfoundland and Labrador; Greenland (an autonomous country within the Kingdom of Denmark); the Faroe Islands; Iceland; the northern regions of Norway, Sweden and Finland; as well as the Russian Arctic regions of Murmansk Oblast, Arkhangelsk Oblast, Nenets, Yamalo-Nenets, Khanty–Mansi and Chukotka Autonomous Okrugs, Komi and Sakha (Yakutia) Republics, Krasnoyarsk Krai, Magadan Oblast and Kamchatka Krai.

A comprehensive estimate of HCV infection prevalence specific to Arctic countries is unavailable. Previous reports summarising the limited available epidemiologic data on HCV infection in Arctic populations indicated that the prevalence of and risk factors for HCV infection were similar to non-Arctic regions of countries [6,7]. The prevalence of HCV infection based on serologic evidence ranges from 0.5 to 2% in Canada, Russia, Alaska (US) and Greenland [6]. Indigenous people comprise ca 10% of the Arctic population [8], with certain groups being potentially disproportionately affected by HCV infection. The serologic prevalence of HCV infection among Canadian Inuit people was 1–18% [6]. Compared with the general US population, American Indian-Alaska Native people had a higher incidence of acute HCV infection and were 17 times more likely to die from a liver-related cause than the general US population [7,9].

Effective programs to identify and treat HCV-infected people can eliminate HCV in a population [10,11]; however, the implementation of such a programme is challenging because the Arctic region is large, sparsely populated and has limited healthcare/public health infrastructure outside major urban population centres [12]. Thus, rural residents may have limited local access to diagnostic tests to confirm HCV infection or to healthcare providers with expertise in managing viral hepatitis or antiviral medications. Furthermore, indigenous populations in the Arctic are more likely to live in regions without ready access to HCV-related care and could experience a disproportional burden of HCV morbidity compared with non-indigenous populations [7]. Arctic countries could identify best practices for tackling these mutual challenges by sharing information on surveillance methods and innovations for delivering healthcare in resource-limited settings.

To date, however, no published reports have summarised the epidemiology of HCV among Arctic countries. Therefore, our objective was to summarise the available surveillance data on HCV infection from Arctic countries (including sub-Arctic regions of countries with large Arctic populations). It was anticipated that this report could help public health stakeholders in participating countries to identify surveillance system strengths that could be implemented across all Arctic regions and to foster collaboration among stakeholders to address surveillance gaps common to all regions.

## Methods

Data collection occurred in two phases. First, a viral hepatitis surveillance systems survey was sent by email to public health agency representatives in participating Arctic countries to assess their viral hepatitis prevention and control activities, with a specific goal to determine which aspects of HCV surveillance were conducted by all countries. The survey responses from the first phase guided the development of a standardised HCV surveillance data collection template for public health representatives in each country to complete.

### Viral hepatitis surveillance systems survey

We developed a viral hepatitis surveillance systems survey to assess the existing viral hepatitis prevention and control programs in Arctic countries. The survey was developed with input from epidemiologists at the US Centers for Disease Control and Prevention’s (CDC) Division of Viral Hepatitis, clinical liver disease specialists at the Alaska Native Medical Center and the viral hepatitis prevention coordinator at the State of Alaska Division of Public Health. The survey focused on acute hepatitis A virus infection, chronic hepatitis B virus infection and HCV infection (survey questions did not distinguish between acute and chronic HCV infection). For each virus, the survey evaluated three areas: vaccination/prevention programmes, surveillance systems and disease registries.

The survey was intended to target public health authorities in nine participating countries with Arctic populations (Denmark was also included, as Greenland is part of the Danish Kingdom and the health systems are tightly connected) [5]. We report data for countries/regions that completed the HCV surveillance data template (Iceland and the Faroe Islands did not participate).

The survey was first sent by email in February 2015 to a single representative who was identified as the point of contact for viral hepatitis prevention and control activities at each of the public health agencies in all Arctic countries and regions (except the Faroe Islands, where we did not identify a respondent point of contact). Representatives who did not respond to the initial survey invitation were solicited for a response in March 2015 and again in April 2015. The survey results indicated that HCV surveillance programs were more comprehensive and more uniformly implemented across Arctic countries and regions than prevention and control programs for hepatitis A or B virus infections. Therefore, we decided to focus the second phase on collecting the available HCV surveillance data.

We developed a HCV surveillance data collection template based on the survey results (Table 1).

**Table 1:** Summary of survey responses for hepatitis C virus prevention and surveillance programmes and policies in Arctic countries/regions, 2015 (n = 7 countries)

	Alaska (US)	Canada	Canadian territories			Greenland	Norway	Sweden	Russia
			Yukon	Northwest Territories	Nunavut				
**Prevention programs/policies**									
Recommendations for screening people at high risk for HCV infection	Y	Y	Y	Y	N	N	Y	Y	Y
Programme to actively identify, contact and screen peoples at high risk for HCV infection	Y	N	Y	Y	N	N	N	Y	Y
**Surveillance programs/policies**									
Healthcare providers mandated to report people identified with HCV infection	Y	Y	Y	Y	Y	Y	Y	Y	Y
Clinical laboratories mandated to report positive test results for HCV infection	Y	Y	Y	Y	Y	N	Y	Y	Y
Case definition for acute HCV infection provided	Y	Y	Y	Y	Y	Y	N	Y	Y
Registry of people with chronic HCV infection	Y^a^	N	Y	Y	N	N	Y	Y	Y^b^

HCV: hepatitis C virus; N: No; US: United States; Y: yes.

^a^Registry available for Alaska Native people, but registry not available statewide.

^b^An electronic registry is implemented in seven of 11 Arctic regions of Russia.

N and Y indicates the absence or presence of programme/policy, respectively.

Denmark, as well as Newfoundland and Labrador in Canada, were not included in this survey.

The HCV cases reported for Canada nationally are inclusive of the reported cases that we present separately for each Canadian territory/province.

### HCV surveillance data collection template

The viral hepatitis surveillance system survey informed our development of a data collection template that requested the following information: (i) surveillance case definitions for acute and chronic HCV infection; (ii) number of cases with acute HCV infection reported during 2012–14; (iii) number of cases with chronic HCV infection reported during 2014; (iv) demographic characteristics of cases with acute and chronic HCV infection and (v) HCV risk factors for people with acute HCV infection. We anticipated that many jurisdictions would not be able to share case-based data because of privacy concerns; therefore, the template requested aggregated data for all survey categories. The data collection template was first sent by email in April 2016.

We subsequently learned that many jurisdictions did not collect data on acute HCV cases. Therefore, we modified the data collection template so people with HCV infection that cannot be classified as acute, chronic or resolved (undifferentiated HCV cases) were grouped with chronic HCV cases and referred to as chronic/undifferentiated cases. In addition, the modified template requested data on HCV risk factors for people with chronic/undifferentiated HCV infection during 2014 (in addition to people with acute HCV infection). The change in the surveillance data collection template to improve data completeness after the initial request for information resulted in variation in the risk factor data reported between countries; countries provided risk factor data for cases with acute HCV reported during 2012–14, chronic/undifferentiated HCV reported during 2012–14 or chronic/undifferentiated HCV reported during 2014.

All participating jurisdictions completed the data collection template by August 2016. Because of the small population sizes of many Arctic regions, we do not report numbers for any categories with ≤ 5 cases in order to maintain confidentiality. Our study did not undergo human subjects ethics review because we did not recruit human subjects or collect any personally identifiable data.

### Rates of acute and chronic/undifferentiated HCV cases

We estimated the population under surveillance in each Arctic country/region by using census data for 2013 or 2014 (Table 2).

**Table 2:** Population under surveillance in each Arctic country/region using census data, 2013 or 2014 (n = 7 countries)

Country/Region	Total population	Population (age category, years)^a^			Male population	Data source (Year)
**Countries**						
Alaska (US)	737,354	207,204 (< 20)	458,910 (20–64)	71,240 (≥ 65)	381,789	Alaska Department of Labour and Workforce Development (2014)
Canada	35,118,845	4,022,389 (< 20)	9,928,894 (20–59)	3,460,602 (≥ 60)	17,411,885	Statistics Canada (2013) http://www.statcan.gc.ca
Denmark	5,627,235	1,250,679 (< 19)	3,349,822 (20–64)	1,026,734 (≥ 65)	2,792,279	Statbank Denmark (2014) http://www.statistikbanken.dk/statbank5a/default.asp?w=1920
Finland	5,471,753	1,203,190 (< 19)	3,177,175 (19–64)	1,091,388 (≥ 65)	2,691,863	Statistics Finland (2014) http://www.stat.fi
Greenland	56,282	16,146 (> 20)	35,894 (20–64)	4,242 (≥ 65)	29,732	Statistics Greenland (2014) http://bank.stat.gl/pxweb/en/Greenland
Norway	5,109,056	1,191,002 (< 19)	3,105,496 (19–64)	812,558 (≥ 65)	2,567,434	Statistics Norway (2014) https://www.ssb.no/en/befolkning
Sweden	9,747,355	2,093,420 (< 19)	5,741,051 (19–64)	1,912,884 (≥ 65)	4,872,240	Statistics Sweden (2014) http://www.scb.se/en
**Canadian territories/province**						
Yukon Territory	37,183	8,186 (< 20)	22,601 (20–59)	6,394 (≥ 60)	18,983	Yukon Bureau of Statistics (2014) http://www.eco.gov.yk.ca/stats/pdf/populationDec_2014.pdf
Northwest Territories	43,980	11,675 (< 19)	29,427 (19–64)	2,878 (≥ 65)	22,469	Northwest Territories Bureau of Statistics (2014) http://www.statsnwt.ca/population/population-estimates
Nunavut Territory	36,083	17,782 (< 25)	16,987 (25–65)	1,314 (≥ 65)	18,665	Nunavut Bureau of Statistics (2014) http://www.stats.gov.nu.ca/en/Population%20estimate.aspx
Newfoundland and Labrador province	529,069	104,428 (< 20)	331,046 (19–64)	93,595 (≥ 65)	260,922	Newfoundland and Labrador Statistics Agency (2014) http://www.stats.gov.nl.ca/statistics/population
**Russian Arctic regions**						
Murmansk Oblast	766,281	165,036	475,497	125,748	366,899	Statistics Russia (2014) http://www.gks.ru
Arkhangelsk Oblast	1,139,950	247,023	661,081	231,846	532,780	
Nenets Autonomous Okrug	43,373	12,472	25,474	5,427	21,126	
Komi Republic	864,424	201,631	524,469	138,324	408,382	
Yamalo-Nenets Autonomous Okrug	539,985	149,496	358,713	31,776	270,704	
Khanty-Mansi Autonomous Okrug	1,612,076	426,454	1,035,084	150,538	785,817	
Krasnoyarsk Krai	2,858,773	647,241	1,709,742	501,790	1,334,600	
Sakha (Yakutia) Republic	956,896	282,005	564,537	110,354	464,570	
Magadan Oblast	148,071	32,432	93,029	22,610	71,715	
Kamchatka Krai	317,269	69,664	198,016	49,589	158,426	
Chukotka Autonomous Okrug	50,540	13,729	32,433	4,378	25,769	

US: United States.

^a^Data reported according to age categories as pre-defined by each country/region that most closely approximate age categories used for present report (< 19 years, 20–64 years and ≥ 65 years).

The numbers reported here come from the national/regional population statistic sources. The age groups available to aggregate did not always line up with the way the surveillance data were provided. Age ranges were selected for the population that aligned best with the surveillance data.

We calculated the rate per 100,000 people of acute and chronic/undifferentiated HCV infections by dividing the number of cases reported by the population in the corresponding country/region. In order to minimise double counting of cases with chronic/undifferentiated HCV infection who might have been reported in multiple years, we restricted our rate estimates to cases reported in a single year (2014 for all countries and regions, except for Canada, for which 2013 data were used). The HCV cases reported for Canada nationally are inclusive of the reported cases that we present separately for each Canadian territory/province. We did not perform statistical tests to compare rates/proportions because the surveillance data were assumed to represent complete counts (no sampling error). To minimise the influence of random variation associated with a small number of observations, we followed the US National Center for Health Statistics practice of not comparing any rates/proportions based on < 20 counts [13].

## Results

A total of seven Arctic and sub-Arctic countries contributed data to this report. The population of the countries that participated in this study ranged from 56,282 (Greenland) to 35,118,845 (Canada) (Table 2). The population of the four territories and one province in Canada ranged from 36,083 (Nunavut) to 529,069 (Newfoundland and Labrador). The population of the 11 subnational Russian Arctic regions ranged from 43,373 (Nenets Autonomous Okrug) to 2,858,773 (Krasnoyarsk Krai).

Among the three countries that reported data on acute HCV infections in 2014 (Table 3), Greenland reported zero cases, Denmark reported seven cases (rate: 0.1/100,000 population) and Sweden reported 143 (rate: 1.5/100,000 population). Of the seven cases in Denmark and 143 in Sweden, 100% and 98% were aged 10–64 years respectively.

**Table 3:** Number and rate of people with acute and chronic/undifferentiated hepatitis C virus infection^a^ reported by Arctic country/region, 2014 (n = 7 countries)

Country/Region	Acute HCV cases		Chronic/undifferentiated HCV cases^b^	
	Number	Rate^c^	Number	Rate^c^
**Countries**				
Alaska (US)	NA	NA	1,262	171.2
Canada (2013)	NA	NA	10,379	29.6
Denmark	7	0.1	234	4.2
Finland	NA	NA	1,224	22.5
Greenland	0	0	0	0
Norway	NA	NA	1,213	23.7
Sweden	143	1.5	1,751	18.0
**Canadian territories/province**				
Yukon	≤ 5	NR	19	51.1
Northwest Territories	NA	NA	17	38.7
Nunavut	NA	NA	6	16.6
Newfoundland and Labrador	NA	NA	128	24.2
**Russian Arctic Regions**				
Murmansk Oblast	10	1.3	369	47.6
Arkhangelsk Oblast	8	0.7	497	43.1
Nenets Autonomous Okrug	0	0.0	22	51.3
Komi Republic	32	3.7	353	40.3
Yamalo-Nenets Autonomous Okrug	17	3.1	434	80.3
Khanty-Mansi Autonomous Okrug	49	3.1	1,075	67.6
Krasnoyarsk Krai	47	1.6	1,668	58.5
Sakha (Yakutia) Republic	10	1.0	373	39.0
Magadan Oblast	≤ 5	NR	52	34.4
Kamchatka Krai	7	2.2	246	76.8
Chukotka Autonomous Okrug	0	0.0	14	27.6

HCV: hepatitis C virus; NA: not available; NR: Not reported.

^a^Undifferentiated HCV infection case defined as a person with acute, chronic or resolved HCV infection; cases might include people reported more than once in 2014 and do not represent unique individuals.

^b^Acute HCV cases for Yukon Territory, Denmark and Sweden are not included among chronic/undifferentiated HCV cases.

^c^Number of cases reported/100,000 people; not calculated for regions with ≤ 5cases.

Categories with 1–5 people (NR) are not reported to maintain confidentiality.

The HCV cases reported for Canada nationally are inclusive of the reported cases that we present separately for each Canadian territory/province.

The rate of reported acute HCV infections in the 11 Russian Arctic regions ranged from 0/100,000 in the Nenets Autonomous Okrug and Chukotka Autonomous Okrug to 3.7/100,000 in the Komi Republic. The Yukon Territory was the only Canadian subnational Arctic region to collect data on acute HCV cases and reported ≤ 5 acute HCV cases in 2014 (rate not calculated). The rate of chronic/undifferentiated HCV cases reported for 2014 in the seven of the Arctic countries ranged from 0/100,000 in Greenland to 171.2/100,000 in Alaska. In the four Canadian territories, it ranged from 16.6/100,000 in Nunavut to 51.1/100,000 in the Yukon territory, and in the 11 Russian Arctic regions it ranged from 27.6/100,000 in Chukotka Autonomous Okrug to 80.3/100,000 in Yamalo-Nenetsk Autonomous Okrug.

Among the Arctic jurisdictions reporting > 20 case-persons with chronic/undifferentiated HCV infection for 2014 (Table 4), the majority (> 80%) of cases were aged 19–64 years and the majority were male in all jurisdictions except for the Russian regions of Murmansk Oblast (46.2%) and Sakha (Yakutia) Republic (38.7%).

**Table 4:** Characteristics of people with chronic/undifferentiated hepatitis C virus infection reported by country/region, 2014^a,b^

Country/Region	n (%) Age years^c^				n (%) sex				Total
	< 19	20–59	> 59	Unknown	Male	Female	Unknown		
**Countries**									
Alaska (US)	8 (0.6)	1,194 (94.6)	59 (4.7)	< 0.01 (1)	690 (54.7)	572 (45.3)	0	1,262	
Canada (2013)	223 (2.3)	8,492 (81.5)	1,377 (15.4)	0.9	6,326 (62.9)	3,757 (36.3)	16 (0.1)	10,379	
Denmark	NR	221 (94.4)	NR	NA	158 (67.6)	76 (32.4)	0	234	
Norway	24 (2.0)	1,115 (91.9)	74 (6.1)	0.0	785 (64.8)	409 (33.8)	19 (1.4)	1,213	
Sweden	27 (1.5)	1,598 (91.3)	126 (7.2)	0.0	1,179 (67.3)	569 (32.5)	3 (0.2)	1,751	
Finland	37 (3.0)	1,164 (95.1)	23 (1.9)	0.0	802 (65.5)	422 (34.5)	0	1,224	
**Canadian territories/province**									
Yukon	NR	17 (89.5)	NR	NA	12 (63.2)	7 (36.8)	NA	19	
Northwest Territories	NA	NA	NA	NA	NA	NA	NA	17	
Nunavut	NR	NR	NR	NA	NR	NR	NA	6	
Newfoundland and Labrador	NR	123 (82.0)	NR	NA	78 (60.9)	50 (39.0)	0	128	
**Russian Arctic Regions^d^**									
Murmansk Oblast	14 (3.8)	336 (91.1)	19 (5.1)	NA	170 (46.2)	199 (53.8)	NA	369	
Arkhangelsk Oblast	12 (2.4)	426 (85.7)	59 (11.9)	NA	259 (52.2)	238 (47.8)	NA	497	
Nenets Autonomous Okrug	NR	18 (81.8)	NR	NA	14 (61.5)	8 (38.5)	NA	22	
Komi Republic	7 (2.0)	324 (91.8)	22 (6.2)	NA	NA	NA	NA	353	
Yamalo-Nenets Autonomous Okrug	5 (1.2)	420 (96.8)	9 (2.1)	NA	NA	NA	NA	434	
Khanty-Mansi Autonomous Okrug	24 (2.2)	969 (90.1)	82 (7.6)	NA	630 (58.6)	445 (41.4)	NA	1,075	
Krasnoyarsk Krai	28 (1.7)	1,520 (91.1)	120 (7.2)	NA	NA	NA	NA	1,668	
Sakha (Yakutia) Republic	10 (2.7)	283 (75.9)	80 (21.4)	NA	144 (38.7)	229 (61.3)	NA	373	
Magadan Oblast	NR	47 (90.4)	NR	NA	31 (58.9)	21 (41.1)	NA	52	
Kamchatka Krai	NR	236 (95.9)	NR	NA	157 (63.8)	89 (36.2)	NA	246	
Chukotka Autonomous Okrug	NR	12 (85.7)	NR	NA	NA	NA	NA	14	

NA: not available; NR: not reported.

^a^Greenland reported zero persons with HCV in 2014 and is not represented in the table. Canadian national HCV surveillance data is presented for 2013 (2014 data were unavailable at the time of this report). Northwest Territories did not provide age/sex of HCV cases.

^b^Undifferentiated HCV infection case defined as individuals with acute, chronic or resolved HCV infection.

^c^Age groups for Russian Arctic and Canada are ages < 20 years, 20–59 years, ≥ 60 years; for Norway they are ages < 19 years, 19–59 years, ≥ 60 years; for Denmark they are ages < 19, 19–64, > 65.

^d^Sex distribution is known for 7 out of 11 regions where the electronic HCV registry is implemented.

Categories with 1–5 people (NR) are not reported to maintain confidentiality.

The HCV cases reported for Canada nationally are inclusive of the reported cases that we present separately for each Canadian territory/province.

Alaska, Nunavut and Denmark were the only three jurisdictions that provided information on whether HCV cases belonged to an indigenous group. Among the 1,262 chronic/undifferentiated HCV cases reported in Alaska during 2014, 179 (14%) persons were indigenous, 858 (68%) persons were non-indigenous and indigenous status was unknown for 225 (18%) persons. Fewer than five indigenous cases with HCV infection were reported in Nunavut and Denmark during 2014.

Information on risk factors for HCV infection was collected by three countries (Norway, Sweden and Denmark), three Canadian regions (Yukon Territory, Northwest Territories, and Newfoundland and Labrador) and all Russian Arctic regions. Injection-drug use was the most frequently reported risk factor in all regions, with > 20 cases, except in the Russian Arctic region, where stigma associated with injection-drug use might have resulted in under-reporting of that risk factor (Table 5).

**Table 5:** Risk factors identified for persons with acute or chronic/undifferentiated hepatitis C virus infection reported by Arctic country/region, 2012–14^a,b^

Risk factors for persons with acute or chronic HCV infection^c^	Country/Region (Year(s))								
	Yukon Territory (2012–14)	Northwest Territories (2012–14)	Newfoundland and Labrador (2014)	Norway (2014)	Sweden (2012–14)		Denmark (2012–14)	Finland (2014)	Russian Arctic regions (2012–14)
	Acute (n < 5)	Chronic (n = 48)	Chronic (n = 128)	Chronic (n = 1,213)	Acute (n = 500)	Chronic^d^ (n = 5,430)	Acute (n = 35)	Chronic (n = 1,224)	Acute^e^ (n = 480)
**Injection-drug use**	**NR**	**20 (42%)**	**47 (37%)**	**641 (53%)**	**311 (62%)**	**2,370 (44%)**	**23 (66%)**	**701 (57%)**	**64 (13%)^f^**
Men who have sex with men	NA	NA	NA	8 (1%)	14 (3%)	31 (1%)	17 (49%)	6 (1%)	0^f^
Suspected/confirmed HCV + heterosexual contact	NR	NR	18 (14%)	31 (3%)	36 (7%)	200 (4%)	NR	57 (5%)	106 (22%)^f^
Suspected/confirmed HCV + household contact	NA	NR	NR	NA	NA	NA	0	NA	23 (5%)
Having undergone surgery	NA	NR	NA	NA	NA	NA	0	NA	NR
Body piercings and tattoos	NA	NR	NA	28 (2%)	9 (2%)	49 (1%)	NR	30 (2%)	18 (4%)^g^
Blood transfusion	NA	NR	6 (5%)	26 (2%)	8 (2%)	216 (4%)	NR	13 (1%)	NR
Maternal to child transmission	NA	NA	NA	NR	NR	30 (1%)	NA	4 (0.3%)	7 (1%)
Nosocomial infection	NA	NA	NA	7 (1%)^h^	NR	66 (1%)^h^	NA	0	13 (3%)^i^
Unknown/missing	0	18 (38%)	NA	488 (40%)	102 (20%)	2,368 (44%)	2 (6%)	413	246 (51%)

HCV: hepatitis C virus; NA: not available; NR: not reported.

^a^Alaska, Canada (national surveillance) and Nunavut surveillance systems do not collect risk factor data on persons reported with HCV infection. Greenland had zero persons with HCV reported in 2014.

^b^Unless otherwise indicated, chronic HCV infection category includes persons with undifferentiated HCV infection, defined as a person who could have acute, chronic or resolved HCV infection; infection could have occurred in a country/region other than where case was detected.

^c^Risk factor information was not uniformly defined or collected between countries/regions; categories might not be mutually exclusive so same case-persons can be represented in multiple risk factor categories.

^d^Includes persons with chronic or undifferentiated HCV infection and excludes persons with acute HCV infection.

^e^Data reported for 11 Russian Arctic regions.

^f^Injection-drug use and men who have sex with men might be under-reported because of stigma with increased false-reporting of heterosexual contact.

^g^Includes other cosmetic procedures such as manicures and pedicures.

^h^Nosocomial infection suspected either abroad or before 1990. No suspected nosocomial transmission in Norway in 2014.

^i^Excluding surgery and blood transfusion.

Categories with 1–5 people (NR) are not reported to maintain confidentiality.

## Discussion

We collated available surveillance data on HCV infection in Arctic populations from seven Arctic countries including three territories and one province in Canada and 11 subnational Arctic regions in Russia. We found that HCV infection was more frequently reported among males, persons aged 19–64 years (20–59 years in the Russian Arctic regions) and individuals with a history of injection-drug use. We could not directly compare the HCV infection rates between countries/regions because of differences in screening practices to identify persons at risk for HCV, as well as differences in surveillance case definitions and reporting of surveillance data (i.e. acute, chronic or undifferentiated HCV). Nevertheless, the data presented in this report can provide insights into the HCV surveillance systems currently operating in the Arctic.

The incidence and prevalence of HCV infection, as measured by any passive surveillance system, can vary greatly according to a number of factors along the HCV continuum of care [11,14]. The prevalence of risk factors for HCV acquisition, such as injection-drug use, and the presence/effectiveness of programs to address HCV risk factors, such as needle-exchange or opiate-substitution therapy programs, will impact the potential number of persons with HCV infection in a population [15]. Before individuals with HCV infection can be reported to a surveillance system, they must be tested; however, many patients are not tested for HCV because they are asymptomatic or are not assessed for HCV risk factors. Consequently, many people are unaware of their infection status [11]. The reporting of HCV cases is, therefore, dependant on testing policies and on the resources available in each country to reach out and screen at-risk persons [16]. After a case is reported to the surveillance system, additional investigation might be necessary to confirm the HCV infection status (acute, chronic or resolved), to identify potential risk factors for infection and to determine whether the case is part of an outbreak requiring public health intervention. Further, identified HCV cases need to be linked to treatment and retained in care until they are cured, which can decrease the prevalence of chronic HCV infection in a population. At each step along the HCV continuum (from screening to treatment/cure) there can be considerable variation between countries [16,17]; HCV infection rates reported by passive surveillance, therefore, do not necessarily represent the true burden of disease in a population and direct comparisons cannot be made between countries/regions.

Despite the limitations associated with interpreting HCV surveillance data, our report can be useful for identifying similarities in HCV surveillance activities between Arctic countries/regions. All Arctic countries/regions collect data on HCV, which indicates that understanding the burden of HCV infections is an important public health priority. All jurisdictions rely on a passive surveillance system to identify HCV cases; most improve the identification of cases by requiring that healthcare providers and laboratories report all individuals that test positive for HCV infection. Many countries/regions also attempt to collect data on risk factors for having HCV infection.

We identified variations in the demographic and epidemiologic characteristics reported for each case between the countries/regions. For example, some countries/regions do not distinguish between acute/chronic/resolved infections or do not remove duplicate HCV cases. Thus, cases may be reported to public health authorities more than once. As another example, the Alaska Department of Health and Human Services does not collect data on risk factors for having HCV, whereas Russia is able to obtain more comprehensive epidemiologic information about each case through the use of an electronic HCV patient registry. Furthermore, the case definition for acute HCV infection and the definitions of the risk factors for having HCV infection are not uniform across jurisdictions; Finland, for example, does not have a surveillance case definition. Finally, few countries/regions report data on persons suspected of having acute HCV infection.

This HCV surveillance report illustrates the known challenges associated with comparing HCV surveillance data between jurisdictions. Modelled estimates of the prevalence of chronic HCV infection in Arctic countries range from 0.8% in Canada to 2.6% in Russia (data available from the Institute for Health Metrics and Evaluation [18]). The modelled chronic HCV prevalence rates are discordant with the annual rate of chronic HCV cases reported by Arctic countries in our study. The differences in the rates of HCV infection between Arctic countries/regions likely reflect differences in the comprehensiveness of screening at-risk populations for HCV infection or differences in access to HCV screening services (especially between rural and urban populations). These factors—not distinguishing acute from chronic HCV infection, potential double-counting of HCV cases and variation in identifying persons with HCV infection—limit direct comparisons of the HCV infection rates we report between countries/regions.

We also identified an important gap in knowledge regarding the epidemiology of HCV in the Arctic. Specifically, most countries/regions do not classify whether HCV cases belong to an officially recognised indigenous or socioeconomic group, so data on potential health disparities related to race/ethnicity or income is lacking. In the US, American-Indian/Alaska Native people have the highest incidence of acute HCV infections of any race/ethnicity [19]. Thus, collecting data on the indigenous status of HCV cases could help to evaluate whether similar disparities exist in other Arctic populations.

Despite the differences in the HCV surveillance systems between countries/regions, there still appears to be some general trends regarding HCV in the Arctic. The leading risk factor for having HCV infection among Arctic populations appears to be injection-drug use, the same as in other non-Arctic populations [20]. Sharing successful practices for reducing the prevalence of injection-drug use or for improving harm reduction strategies could provide an opportunity for collaboration between Arctic countries/regions. In addition, the majority of persons with chronic/undifferentiated HCV infection were within the broad age category of 19–64 years. Although all regions collect the specific ages of reported cases, we were unable to present data by more precise age groups because of privacy concerns. Assuming that the prevalence of HCV infection in the Arctic is similar to that estimated for other high-income countries, we would expect the prevalence to be highest among adults aged 45–64 years [21]. In addition, if the rates of injection-drug use are higher among young adults, as observed in other high-income regions, we would expect the incidence of HCV to be high among young adults as well [22,23].

Ideally, HCV surveillance data from Arctic countries/regions should be summarised on a regular basis to allow for monitoring relative disease trends. Future surveillance reports could be improved if the participating public health agencies collaborated to develop standardised case definitions (including definitions for HCV risk factors) and data reporting templates. Demographic and risk factor information on HCV cases could be obtained more thoroughly and efficiently by linking HCV surveillance databases with those for other blood-borne pathogens, such as human immunodeficiency virus. To facilitate the development of standardised HCV surveillance protocols, public health authorities from Arctic countries could consider developing a position statement on conducting surveillance for HCV infections. The statement could address the unique surveillance challenges confronting low- or middle-income (typically rural) regions within high-income countries. Uniform reporting of surveillance data could help to make more meaningful comparisons of HCV epidemiology between regions. Comparing trends in the epidemiology of HCV infections between regions could help to identify successful prevention and control programs, as well as policies that could be considered for implementation in other regions.
